# Developing a Prognostic Micro-RNA Signature for Human Cervical Carcinoma

**DOI:** 10.1371/journal.pone.0123946

**Published:** 2015-04-16

**Authors:** Christine How, Melania Pintilie, Jeff P. Bruce, Angela B. Y. Hui, Blaise A. Clarke, Philip Wong, Shaoming Yin, Rui Yan, Daryl Waggott, Paul C. Boutros, Anthony Fyles, David W. Hedley, Richard P. Hill, Michael Milosevic, Fei-Fei Liu

**Affiliations:** 1 Ontario Cancer Institute, University Health Network, Toronto, ON, Canada; 2 Department of Medical Biophysics, University of Toronto, Toronto, ON, Canada; 3 Division of Biostatistics, Princess Margaret Cancer Centre, University Health Network, Toronto, ON, Canada; 4 Department of Pathology, University Health Network, Toronto, ON, Canada; 5 Department of Radiation Oncology, Princess Margaret Cancer Centre, University Health Network, Toronto, ON, Canada; 6 Department of Radiation Oncology, University of Toronto, Toronto, ON, Canada; 7 Informatics & Biocomputing Platform, Ontario Institute for Cancer Research, Toronto, ON, Canada; 8 Department of Pharmacology and Toxicology, University of Toronto, Toronto, ON, Canada; 9 Division of Medical Oncology, Princess Margaret Cancer Centre, University Health Network, Toronto, ON, Canada; Georgetown University, UNITED STATES

## Abstract

Cervical cancer remains the third most frequently diagnosed and fourth leading cause of cancer death in women worldwide. We sought to develop a micro-RNA signature that was prognostic for disease-free survival, which could potentially allow tailoring of treatment for cervical cancer patients. A candidate prognostic 9-micro-RNA signature set was identified in the training set of 79 frozen specimens. However, three different approaches to validate this signature in an independent cohort of 87 patients with formalin-fixed paraffin-embedded (FFPE) specimens, were unsuccessful. There are several challenges and considerations associated with developing a prognostic micro-RNA signature for cervical cancer, namely: tumour heterogeneity, lack of concordance between frozen and FFPE specimens, and platform selection for global micro-RNA expression profiling in this disease. Our observations provide an important cautionary tale for future miRNA signature studies for cervical cancer, which can also be potentially applicable to miRNA profiling studies involving other types of human malignancies.

## Introduction

Micro-RNAs (miRNAs) are a class of small, non-coding RNAs that play important roles in regulating target genes by binding to complementary sequences in mRNA transcripts [1]. Deregulation of miRNA expression has been reported for numerous solid and hematological malignancies, which is not surprising given their involvement in multiple critical biological processes, including development, proliferation, and apoptosis [2, 3]. miRNAs have been described to be extremely stable, and can be readily extracted from cell lines and various types of clinical specimens, including frozen and formalin-fixed paraffin embedded (FFPE) tissues, blood, serum, plasma, urine, and saliva [4–9]. miRNA expression profiling of solid and hematological human malignancies has identified disease-specific miRNA signatures associated with diagnosis, progression, staging, prognosis and response to therapy [10]. Numerous prognostic miRNA signatures have since been described for various malignancies, including: lung [11–13], colorectal [14], gastric [15], esophageal [16], hepatocellular [17], prostate [18], nasopharyngeal [19], and cervical cancers [20].

Cervical cancer is the third most frequently diagnosed cancer, and the fourth leading cause of cancer mortality in women worldwide, with an estimated 530,000 new cases and 175,000 deaths each year [21]. Although cervical cancer incidence and mortality have declined over the past 30 years in the United States [22], the 5-year survival rate remains less than 40% for patients diagnosed with Stage III disease and above [23]. A number of groups have investigated the role of miRNAs in cervical cancer, including Wang *et al*., who reported that miR-143 and miR-145 suppressed cell growth, whereas miR-146a promoted cell proliferation in cervical cancer [24]. Li *et al*. identified miR-29 to be the most highly enriched HPV-associated miRNA in cervical cancer, which functions by restraining cell cycle progression and inducing apoptosis via YY1 and CDK6 [25]. More recently, Wang et al. reported that oncogenic HPVs induce aberrant expression of host cell miRNAs, but do not produce any detectable viral miRNA [26]. A two-miRNA signature that could predict overall survival in cervical cancer patients was reported by Hu *et al*. [20], and remains the only reported miRNA signature for cervical cancer to date. This signature, which consisted of miR-200a and miR-9, was developed using a qRT-PCR assay measuring 96 cancer-related miRNAs in a training cohort of 60 cervical cancer patients, and was internally validated in a testing cohort of 42 patients. However, we were unable to independently corroborate this signature with our own cohort of patients. Given these limitations and the poor survival for patients with advanced stages of cervical cancer, we sought to develop an independent miRNA signature that could predict disease-free survival (DFS) for cervical cancer patients; and potentially allow tailoring of treatment according to risk. Herein, we describe the challenges and considerations associated with developing such a prognostic miRNA signature for cervical cancer.

## Materials and Methods

### Ethics Statement

Written informed consent was obtained from all human subjects, according to a protocol approved for this study by the University Health Network Research Ethics Board.

### Clinical specimens

Pre-treatment cancer samples were collected from patients with cervical cancer prior to undergoing curative chemo-radiation, consisting of external-beam radiotherapy to the primary cervical tumour and pelvic lymph nodes (45 to 50 Gy total, in 1.8-to-2-Gy daily fractions using 18 or 25MV photons), combined with weekly cisplatin (40 mg/m^2^ total, 5 doses). Patients were staged using the FIGO (International Federation of Gynecologists and Obstetricians) system, with additional clinical information gathered using computed tomography (CT) scans of the abdomen and pelvis and magnetic resonance imaging (MRI) of the pelvis to assess local and lymphatic disease. Pelvic and para-aortic lymph nodes were classified as positive for metastatic disease if the MRI short-axis dimension was >1 cm, and equivocal if it was 8 to 10 mm.

The training cohort comprised of flash-frozen punch biopsies obtained from 79 patients treated at the Princess Margaret Cancer Centre between 2000 to 2007, inclusively. The biopsy specimens were placed in a storage medium (optimal cutting temperature (OCT) compound) for histopathologic examination, then flash-frozen in liquid nitrogen. H&E-stained tissue sections were cut from the OCT-embedded material, and evaluated by a gynecological pathologist (B. Clarke). The total cell content (stroma and tumour cells) was estimated for all tissue samples using a light microscope, and only samples containing at least 70% tumour cells were considered for further analysis. Flash-frozen normal cervix tissues obtained from 11 patients who underwent total hysterectomy for benign causes served as the normal comparators. The clinical data for the patients in the training cohort are found in S1 Table.

The validation cohort comprised of diagnostic FFPE blocks collected from 87 cervical patients treated between 1999 and 2007, inclusively. There was no overlap of patients between the training and validation cohorts. All samples contained at least 70% malignant epithelial cells, as determined by a gynecologic pathologist (B. Clarke), or were macro-dissected prior to RNA purification. FFPE normal cervix tissues obtained from 9 patients who underwent hysterectomy for benign causes served as the normal comparators. The clinical data for the patients in the validation cohort are found in S2 Table.

### Sample processing

For the training cohort specimens, two sections of 50-μm thickness were cut from the OCT-embedded flash-frozen tissues and placed in a nuclease-free microtube. Total RNA was isolated using the Norgen Total RNA Purification Kit (Norgen Biotek), according to the manufacturer’s instructions. Global miRNA expression was measured in both the cervical cancer and normal cervix tissues with the TaqMan Low Density Array (TLDA) Human MicroRNA A Array v2.0 (Applied Biosystems) using the Applied Biosystems 7900HT Real-Time PCR System, as previously described [4].

For the validation cohort specimens, ten sections of 5-μm thickness were cut from the FFPE tissues and placed in a nuclease-free microtube. Total RNA was isolated using the Norgen Total RNA Purification Kit (Norgen Biotek), according to the manufacturer’s instructions. We measured the expression of the 9 miRNAs in our prognostic signature using three methods: 1) Applied Biosystems TLDA Human MicroRNA A Array v2.0 with 300 ng of total RNA per sample; 2) NanoString nCounter Human miRNA Expression Assay v1.6.0 with 200 ng of total RNA per sample; and 3) individual single-well qRT-PCR using Applied Biosystems TaqMan MicroRNA Assays with 10 ng of total RNA per sample, as previously described [4]. The TaqMan MicroRNA Assay includes a reverse transcription step wherein a stem-loop reverse transcription primer specifically hybridises with its target miRNA molecule, which is then reverse-transcribed with a MultiScribe reverse transcriptase. Briefly, each reverse transcription reaction contained 50 nM stem-loop reverse transcription primer, 1x reverse transcription buffer, 0.25 nM dNTPs, 3.33 U/μl MultiScribe reverse transcriptase, 0.25 U/μl RNase inhibitor, and 10 ng total RNA. This reverse transcription reaction was incubated in an Applied Biosystems 7900 Thermocycler for 30 min at 16°C, 30 min at 42°C, 5min at 85°C, and then held at 4°C. The product of this reverse transcription reaction was then amplified with miRNA-specific primers using the Applied Biosystems 7900HT Real-Time PCR system. The PCR reaction consisted of 0.67 μl reverse transcription product, 1x TaqMan Universal PCR Master Mix, and 1x TaqMan miRNA assay. The reactions were loaded onto a 384-well plate and incubated at 95°C for 10 min followed by 40 cycles of 95°C for 15 s and 60°C for 1 min.

### Data normalization

To normalize the miRNA expression data from TLDA, the raw miRNA abundances were loaded into the R statistical environment (v2.15.2). Three control genes were utilized for normalization: RNU44, RNU48, and U6. Normalized miRNA abundances were calculated as -log_2_(2^-(*C_T_-C_C_*)^), where C_T_ represents the threshold cycle, and C_C_ represents the mean threshold cycle of the control genes.

To normalize the miRNA expression data from NanoString, the R package ‘NanoStringNorm’ [27] was utilized with the following settings:

1. Probe level correction.
2. Code Count Correction = "geo.mean" (geometric mean)
3. Background Correction = "mean.2sd", i.e. mean +/- 2 standard deviations (Background is calculated based on negative controls, the calculated background is subtracted from each sample)
4. Sample Content Correction = "top.geo.mean" (The option 'top.geo.mean' is a method which ranks miRNAs based on the sum of all samples and then takes the geometric mean of the top 75)
5. log_2_ transformation

### Survival analysis

The normalized data were filtered to remove miRNAs with low expression (C_t_ > 35) in over 20% of samples. Least Absolute Shrinkage and Selection Operator (LASSO) regression was applied to the normalized TLDA miRNA expression data for the training cohort [28], to select a subset of miRNAs associated with DFS. The parameter was obtained through cross-validation (0.087942). LASSO regression is a method used for variable selection and shrinkage in Cox’s proportional hazards model. This technique shrinks the coefficients and allows some of them to reach zero, which reduces the estimation variance while producing an interpretable final model. The DFS estimates were calculated based on the Kaplan-Meier method. The hazard ratios were obtained from the unadjusted Cox regression method; miRNAs significant for DFS were selected, leading to a 9-miRNA signature prognostic for DFS. The expression of the nine miRNAs were validated in the training cohort and normal control group with individual single-well qRT-PCR using Applied Biosystems TaqMan MicroRNA Assays.

A risk score was calculated using the coefficients obtained in LASSO regression and the normalized miRNA expression levels. The risk scores were dichotomized at the median, and the cohort was divided into low and high risk groups. Kaplan-Meier survival analysis was used to illustrate the difference in DFS between the high and low risk groups. Validation was also attempted by dividing the validation cohort into three groups based on risk scores: high-risk, medium-risk, low-risk). The p-value associated with the two curves was determined using the log-rank test. All statistical analyses were performed with R statistical environment (v2.15.2) using the “Survival” package for survival analysis, and “glmnet” for LASSO analysis. Significance was defined as p-values below 0.05.

Publically-available cervical cancer Illumina Small RNA-Seq data from The Cancer Genome Atlas (TCGA) Data Portal was utilized as another independent validation cohort (n = 48). Level three Small RNA-Seq (isoform_expression) data and clinical information for the 48 cervical cancer patients were downloaded from the Broad Firehose (stddata run 2013_06_06). Reads-per-million (RPM) data were log2 transformed and z-score standardized before the 9-miRNA signature equation was applied to calculate risk scores. Patients were dichotomized by the previously established cut-point (median risk score in training cohort) and compared using a log-rank test.

## Results

### The majority of significantly differentially-expressed miRNAs were downregulated

The clinical characteristics of the patients in the training (n = 79) and validation (n = 87) cohorts are provided in Table 1. Analysis of tumour-normal (T/N) fold changes revealed that the majority of significantly differentially-expressed miRNAs (*P < 0*.*01*) were downregulated in our cervical cancer samples (S3 Table). Of the 29 significantly differentially-expressed miRNAs, only 2 were upregulated (miR-21 and miR-187).

**Table 1:** Clinical parameters of patients in the training and validation cohorts.

	*Training cohort*	*Validation cohort*	
	*(n = 79)*	*(n = 87)*	
Age (years)			P = 0.95
Median	48	48	
Range	26–84	19–83	
Tumour size			P = 0.29
≤ 5 cm	48 (61%)	43 (52%)	
> 5 cm	31 (39%)	39 (48%)	
FIGO stage			P = 0.28
IB	24 (30%)	22 (25%)	
IIA	2 (3%)	5 (6%)	
IIB	35 (44%)	31 (36%)	
IIIA	0	2 (2%)	
IIIB	18 (23%)	27 (31%)	
Pelvic or para-aortic node involvement			P = 0.91
Positive	25 (32%)	29 (33%)	
Equivocal	15 (19%)	18 (21%)	
Negative	39 (49%)	40 (46%)	
Overall survival			P = 0.94
Deaths	24 (31%)	26 (30%)	
Disease-free survival			P = 0.74
Relapses or deaths	28 (35%)	33 (38%)	
Follow-up (years)			
Median	6.0	5.3	
Range	0.7–10.6	1.0–10.5	

### Patient miRNA expression was associated with disease-free survival

Using LASSO regression on the normalized TLDA data from the training cohort (S4 Table), we derived the following model to calculate the risk score for each patient using the expression values of nine miRNAs:

$$\text{Risk Score }=\left(0.197109\text{x }{\text{E}}_{\text{let}-7\text{c}}\right)–\left(0.07048\text{x }{\text{E}}_{\text{miR}-21}\right)+\left(0.045797\text{x }{\text{E}}_{\text{miR}-222}\right)–\left(0.56469\text{x }{\text{E}}_{\text{miR}-451}\right)+\left(0.171838\text{x }{\text{E}}_{\text{miR}-455-5\text{p}}\right)–\left(0.01725\text{x }{\text{E}}_{\text{miR}-134}\right)+\left(0.506956\text{x }{\text{E}}_{\text{miR}-148\text{a}}\right)+\left(0.203466\text{x }{\text{E}}_{\text{miR}-218}\right)+\left(0.22355\text{x }{\text{E}}_{\text{miR}-500}\right)$$

Where E_X_ represents the normalized expression level of X, and X represents one of the nine miRNAs in the prognostic signature.

**Fig 1:**
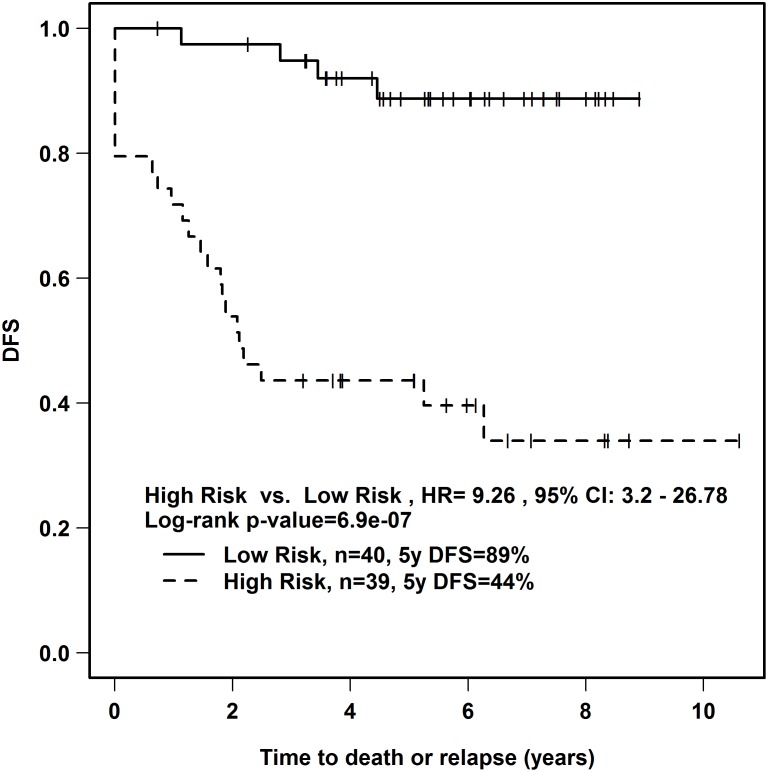
Kaplan-Meier analysis of DFS according to 9-miRNA signature. A risk score was calculated for each patient in the training cohort (n = 79) using our 9-miRNA signature for DFS in cervical cancer. The median risk score was used to divide patients into the high *vs*. low risk groups. HR; hazard ratio, DFS; disease-free survival, CI; 95% confidence interval.

In this prediction model, a higher risk score would predict for poorer DFS. The risk score was calculated for each patient in the training set, and the median risk score (-0.05373) was used to dichotomize the low *vs*. high risk groups. Our 9-miRNA signature was significantly predictive of DFS for the 79 patients in the training cohort, with a hazard ratio of 9.26 and log-rank p-value of 6.9 x 10^-7^ (Fig 1), and was independent of FIGO stage, tumour size and nodal status.

### Validation of miRNA signature

**Fig 2:**
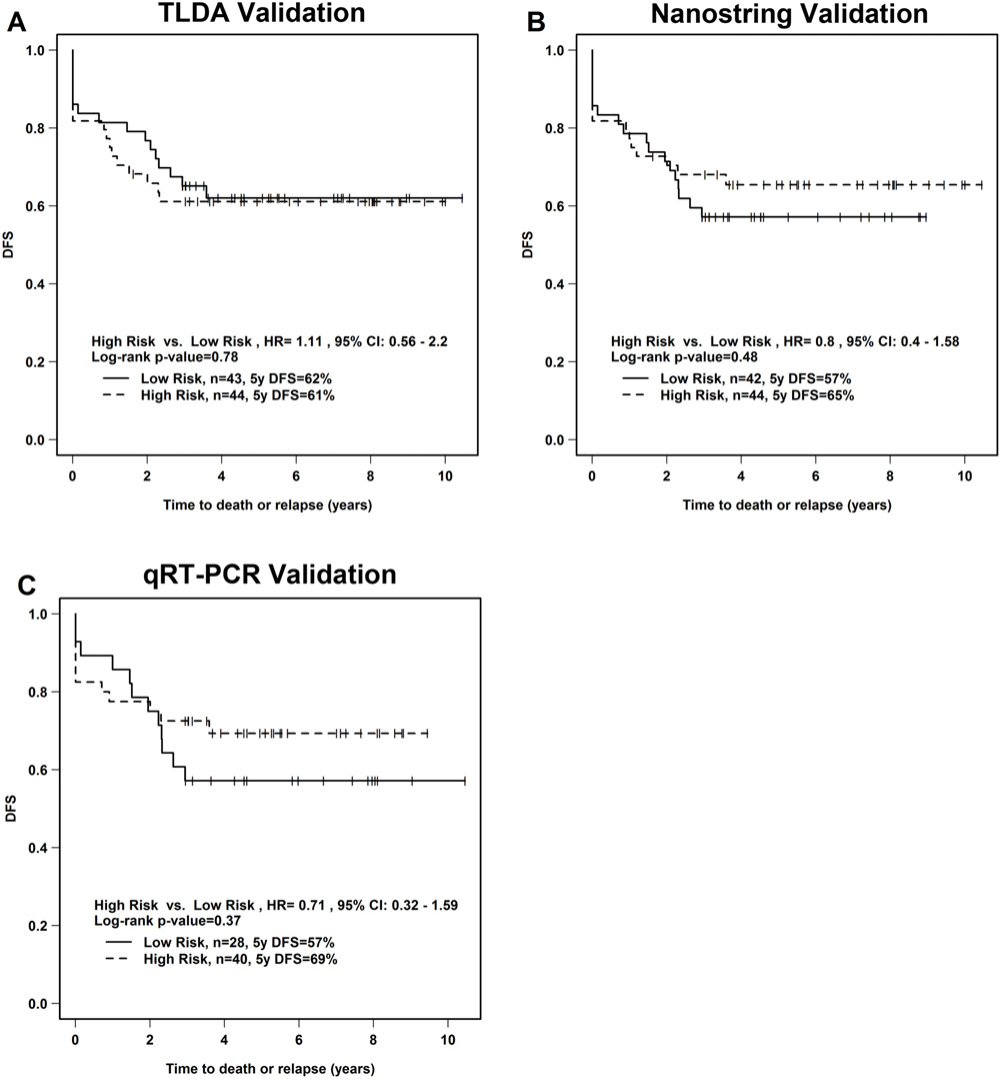
Application of 9-miRNA signature to validation cohort. Kaplan-Meier analysis of DFS. A risk score was calculated for each patient in the validation cohort, by applying our 9-miRNA signature for DFS to the miRNA expression data generated using A) TLDA, B) NanoString, and C) individual qRT-PCR. The same cut-off point from the training set was used. HR; hazard ratio, DFS; disease-free survival, CI; 95% confidence interval.

In our attempts to validate the 9-miRNA signature, we used three different methods to measure miRNA expression in the validation cohort: 1) TLDA (n = 87); 2) NanoString (n = 87); and 3) qRT-PCR (n = 68; 19 samples omitted due to insufficient RNA). A risk-score was calculated for each of the patients by applying the miRNA TLDA expression values (S5 Table) to our prediction model. The patients were divided into the high *vs*. low risk groups based on their risk score, using the same cut-off point as defined in the training cohort (Fig 2A). This analysis was repeated for the NanoString (S6 Table) and the qRT-PCR (S7 Table) datasets (Fig 2B and 2C). Regardless of the method used for measuring miRNA expression, the 9-miRNA signature was not significant when applied to the patients in the validation cohort. In addition, dividing the validation cohort into three groups based on risk scores (high-risk, medium-risk, low-risk) did not lead to a significant result in the TLDA, NanoString or qRT-PCR datasets (S1 Fig). We also utilized publically-available cervical cancer Illumina Small RNA-Seq data derived from The Cancer Genome Atlas (TCGA) Data Portal as another independent validation cohort (n = 48). Interestingly, this validation attempt approached statistical significance (p = 0.05251) (S2A Fig).

### Independent Corroboration of the Hu et al. 2-miRNA signature

**Fig 3:**
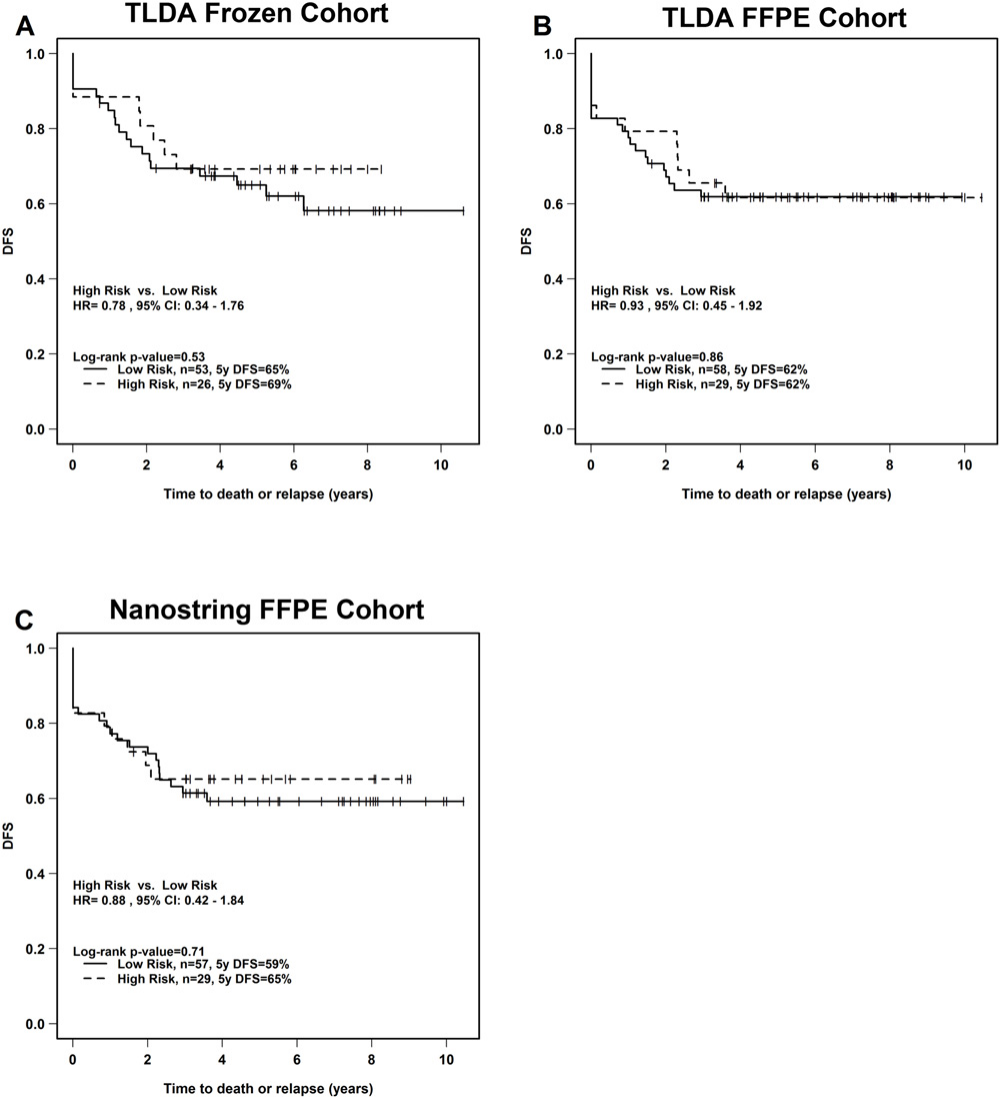
Kaplan-Meier analysis of DFS according to Hu *et al*. 2-miRNA signature. Using the Hu *et al*. 2-miRNA signature, a risk score was calculated for each patient from: A) TLDA frozen cohort (n = 79), B) TLDA FFPE cohort (n = 87), and C) NanoString FFPE cohort (n = 87). HR; hazard ratio, DFS; disease-free survival, CI; 95% confidence interval.

We attempted to perform an independent corroboration of the only published prognostic miRNA signature to date for cervical cancer by Hu, *et al*. (S = 17.9 − 0.284 × EmiR-9 − 0.376 × EmiR-200a, where S represents the risk score for each patient, and EmiR-9 and EmiR-200a represent the normalized expression levels of miR-9 and miR-200a in each patient, respectively) [20]. This 2-miRNA signature was first applied to the miRNA TLDA expression values from our training frozen samples (n = 79) to calculate a risk-score for each patient. Although the authors used 0 as the cut-off point with their training set, we were not able to use this value for corroboration because the risk scores were all above 0. We thus divided our patients into high *vs*. low risk groups, with 1/3 in the former and 2/3 in the latter categories, which reflected the Hu *et al*. population when 0 was used as their cut-off. Based on the Kaplan-Meier analysis, the 2-miRNA signature was not significant when applied to the patients in our frozen cohort utilizing the TLDA platform (Fig 3A). This analysis was repeated for the FFPE TLDA, and FFPE NanoString datasets; neither of which could corroborate the 2-miRNA signature (Fig 3B and 3C). In a final attempt to corroborate the Hu *et al*. signature, we utilized Small RNA-Seq data derived from the TCGA Data Portal as yet another independent cohort (n = 48). Using the same analytical methods, again, the 2-miRNA signature could not be corroborated (S2B Fig), although at least the trend was in the correct direction, in contrast to the other 3 datasets.

An attempt was also made to cross-validate our own 9-miRNA signature with the miRNA expression data from the Hu, *et al*. study; unfortunately, access to their raw data was not provided; hence no further analysis was possible.

## Discussion

In the clinical management of cancer patients, prognostic evaluation is essential to guide appropriate treatment decisions. Unfortunately, in cervical cancer, there remains significant differences in patient survival despite being assigned to the same clinical stage; underscoring the gaps in the current system, as well as the need to develop more useful prognostic biomarkers. We developed a candidate 9-miRNA signature that was prognostic for DFS in patients with cervical cancer. A number of the miRNAs in this signature have been previously characterized in cervical cancer and other diseases, such as miR-218, which downregulates survivin (BIRC5) in nasopharyngeal carcinoma [29], and miR-21, which regulates PTEN expression in hepatocellular carcinoma [30]. According to our analysis, our validation cohort (n = 87) was large enough to detect a signature with a HR of 2.5 with 83% power. However, we could not validate our signature (HR = 9.26) in our validation cohort, despite trying three separate techniques. Furthermore, attempts to corroborate the only published miRNA signature to date for cervical cancer [20] in three independent patient cohorts were also unsuccessful.

We believe that there are three key reasons as to why we could not validate our candidate 9-miRNA prognostic signature for cervical cancer: i) intra-tumour heterogeneity; ii) miRNA expression data from frozen and FFPE samples could not be directly compared in this disease; and iii) the current platforms for global miRNA expression profiling are not sufficiently robust.

Firstly, it is well-established that human tumours are intrinsically heterogeneous. Two types of tumour heterogeneity exist: a) inter-tumour heterogeneity, with differences between tumours arising from different patients; and b) intra-tumour heterogeneity, with differences between distinct sub-populations of cancer cells within a single individual’s tumour. In cervical cancer, intra-tumour heterogeneity has been described on various levels. Many studies have demonstrated significant variations in interstitial fluid pressure [31], blood perfusion [32], and oxygen tension [33] in different regions within an individual tumour. At the chromosomal level, intra-tumour heterogeneity has been described with respect to specific genetic mutations and chromosomal abnormalities such as gains and deletions, reflecting the polyclonal derivation of cervical cancer [34–38]. At the mRNA transcript level, Bachtiary *et al*. evaluated intra-tumoural heterogeneity across 11 cervical cancer patients, and demonstrated that multiple biopsies from distinct areas of an individual tumour were necessary to reduce sampling bias, with genes displaying low intra-tumour heterogeneity requiring two to three biopsies, and genes with high intra-tumour heterogeneity requiring more than six biopsies per tumour [39].

To date, intra-tumoural heterogeneity of miRNA expression has only been reported in breast cancer thus far [40]. However, natural inter-patient variability has been shown to exist among normal cervix samples, which complicates miRNA expression profiling studies [41]. Given that intra-tumour heterogeneity exists in cervical cancer at the macroscopic, chromosomal and transcript levels, it would be a logical extension to assume that this would also apply to miRNAs. In our study, we only utilized one biopsy from each patient in the training and validation cohorts, which is therefore probably insufficient to obtain a representative measure of miRNA expression for the patient’s entire tumour.

A second reason for the lack of signature validation is the lack of concordance with respect to the tissue preservation method used for the two patient cohorts; specifically, the training and validation sets consisted of frozen and FFPE samples, respectively. This experience would appear to contradict several previous studies that have reported high concordance in the miRNA expression data from tissue-matched frozen and FFPE samples derived from various types of human tissues, including: breast [4], lung [42], kidney [43], skin [44], glioblastoma [45], melanoma [46, 47], prostate [48, 49], and lymph nodes [50]. However, with the exception of our own single study that used the TLDA platform [4], the remaining reports utilized other miRNA profiling technologies, such as microarray [42, 44, 46, 47, 50], deep sequencing [42, 43], custom PCR array [48], and qRT-PCR using stem-loop [49] or locked-nucleic-acid primers [45]. There has only been one report to date that evaluated miRNA expression data from tissue-matched frozen and FFPE cervix samples, which only analyzed 3 cervix specimens, in addition to 3 breast and 2 gall bladder samples [51]. This report by Doleshal *et al*. analyzed 3 miRNAs (miR-24, miR-103, miR-191) using qRT-PCR, and only calculated the ΔCt values between frozen and matched FFPE samples without performing any correlation tests. Furthermore, several of these published reports have demonstrated that although there were high correlations (> 0.5) between tissue-matched frozen and FFPE samples for the overall panel of miRNAs tested, there were specific individual miRNAs that were poorly correlated between the matched samples [43, 45, 47], and sometimes even miRNAs that demonstrated opposing patterns of under- or over-expression in tissue-matched sample pairs [48, 49]. The reasons behind these discrepancies, as to why some miRNAs correlate between frozen and FFPE samples; yet others do not, remain unclear.

Lastly, we believe that the current methods used for global miRNA expression profiling are not sufficiently robust. We used the TLDA and NanoString platforms for global miRNA expression profiling, which are both widely-used for miRNA expression profiling. However, in our own recent analyses, the correlation in miRNA expression levels between these two platforms, even using the same FFPE RNA samples, had only a ρ of 0.65 (manuscript in preparation), underscoring the challenges in the technologies to be able to reliably identify clinically useful biomarkers in this disease. This might also provide one technical explanation for the recent review describing the difficulties in validating miRNAs for human malignancies [52]. Given that the majority of miRNAs that were detected in our training cohort by TLDA were expressed at low levels, the detection accuracy may have been limited in this study. Deep sequencing may likely be a more suitable platform to identify and validate a prognostic miRNA signature, since more recent reports have demonstrated the advantages of deep sequencing, including increased sensitivity and specificity with few false-positive calls [53, 54].

Our validation attempt using Small RNA-Seq data from the TCGA data portal was promising, with our 9-miR signature predicting worse outcome for high-risk patients than low-risk patients (p = 0.053). Interestingly, the datasets from the TCGA cohort and our training cohort were both generated from frozen tissues. With the addition of more cervical cancer samples to the TCGA data portal with survival information and miRNA expression data, we could potentially obtain a statistically significant result in a future validation attempt.

In conclusion, a prognostic miRNA signature for cervical cancer could not be validated, due to intra-tumoural heterogeneity, incompatibilities between miRNA expression data from frozen and FFPE samples, and insufficiently robust technical platforms for global miRNA expression profiling. We propose that this could potentially be resolved in the near future, with the advancement of technologies for miRNA expression profiling such as deep-sequencing. At present, the TCGA Data Portal contains deep sequencing miRNA expression data with clinical annotation for only 48 cervical cancer patients; when this dataset is expanded to include more samples, it could potentially be utilized as an important resource to identify and corroborate potential miRNA signature sets. Our observations provide an important cautionary tale for future miRNA signature studies for cervical cancer, which can also be potentially applicable to miRNA profiling studies involving other types of human malignancies.

## Supporting Information

S1 Table Clinical Data, Training Cohort. Clinical data for 79 frozen cervix samples.
(TXT)

S2 Table Clinical Data, Validation Cohort. Clinical data for 87 FFPE cervix samples.
(XLSX)

S3 Table Significantly differentially-expressed miRNAs in cervical cancer. Fold changes (log_2_) of 29 miRNAs that were significantly differentially-expressed in cancer *vs*. normal cervix samples, in order of increasing P-value.
(PDF)

S4 Table TLDA Data, Training Cohort. miRNA expression in 79 frozen cervix samples.
(XLSX)

S5 Table TLDA Data, Validation Cohort. miRNA expression in 87 FFPE cervix samples.
(TXT)

S6 Table NanoString Data, Validation Cohort. NanoString data for 87 FFPE cervix samples.
(TXT)

S7 Table PCR Data, Validation Cohort. PCR data for 68 FFPE cervix samples.
(XLS)

S1 Fig Application of 9-miRNA signature to validation cohort divided into three risk groups. Kaplan-Meier analysis of DFS. A risk score was calculated for each patient in the validation cohort by applying our 9-miRNA signature for DFS to the miRNA expression data generated using A) TLDA, B) NanoString, and C) individual qRT-PCR. The validation cohort was divided into three groups based on risk scores: high-risk, medium-risk, and low-risk. HR; hazard ratio, DFS; disease-free survival, CI; 95% confidence interval.
(TIF)

S2 Fig Application of TCGA Small RNASeq data to cervical cancer miRNA signatures. miRNA expression data from the TCGA miRNASeq cohort (n = 48) was used to test: A) our 9-miR signature, and B) the Hu *et al*. 2-miR signature. HR; hazard ratio, CI; 95% confidence interval.
(PDF)
