# Supplementary material for: Developing a Prognostic Micro-RNA Signature for Human Cervical Carcinoma
Source: PLoS One. 2015 Apr 16;10(4):e0123946. doi: 10.1371/journal.pone.0123946 (PMC4399941; doi:10.1371/journal.pone.0123946)
Supplement: S3 Table — Fold changes (log2) of 29 miRNAs that were significantly differentially-expressed in cancer vs. normal cervix samples, in order of increasing P-value. (PDF) [file pone.0123946.s003.pdf]

S3 Table. Significantly differentially-expressed miRNAs in cervical cancer

miRNA	Fold Change (log_2)	P-value
miR-149	-4.30	2.09×10^{-9}
let-7c	-3.42	5.69×10^{-7}
miR-218	-3.79	5.14×10^{-6}
miR-139-5p	-2.75	1.44×10^{-5}
miR-203	-3.43	1.44×10^{-5}
miR-125b	-2.80	1.44×10^{-5}
miR-376c	-3.08	4.77×10^{-5}
miR-361-5p	-3.76	1.15×10^{-4}
miR-320a	-1.07	1.33×10^{-4}
miR-324-3p	-1.85	1.67×10^{-4}
miR-196b	-1.59	2.07×10^{-4}
let-7b	-1.48	4.24×10^{-4}
let-7a	-1.84	4.24×10^{-4}
miR-100	-2.61	4.24×10^{-4}
let-7e	-1.29	1.16×10^{-3}
miR-193b	-1.14	1.23×10^{-3}
miR-99a	-2.86	1.32×10^{-3}
miR-328	-1.84	1.35×10^{-3}
miR-195	-1.82	1.35×10^{-3}
miR-21	1.89	1.35×10^{-3}
miR-24	-1.10	1.43×10^{-3}
miR-574-3p	-1.57	2.40×10^{-3}
miR-375	-5.72	2.43×10^{-3}
miR-148a	-2.22	2.43×10^{-3}
miR-29a	-1.51	2.70×10^{-3}
let-7g	-0.91	6.46×10^{-3}
miR-210	-1.70	6.80×10^{-3}
miR-455-5p	-2.26	8.50×10^{-3}
miR-187	2.85	8.70×10^{-3}
