# Supplementary figures and images for: Developing a Prognostic Micro-RNA Signature for Human Cervical Carcinoma
Source: PLoS One. 2015 Apr 16;10(4):e0123946. doi: 10.1371/journal.pone.0123946 (PMC4399941; doi:10.1371/journal.pone.0123946)

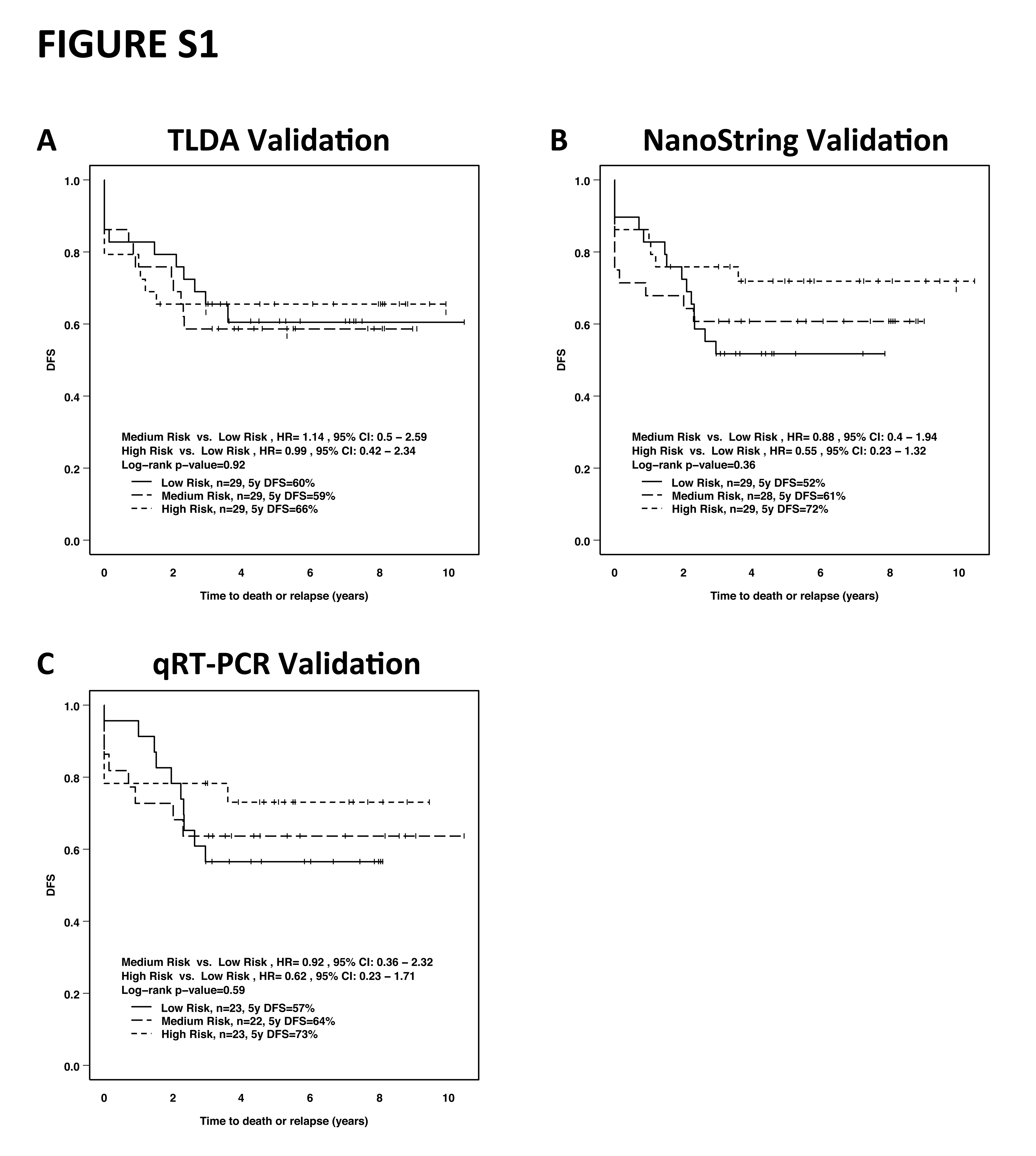

Supplement: S1 Fig — Kaplan-Meier analysis of DFS. A risk score was calculated for each patient in the validation cohort by applying our 9-miRNA signature for DFS to the miRNA expression data generated using A) TLDA, B) NanoString, and C) individual qRT-PCR. The validation cohort was divided into three groups based on risk scores: high-risk, medium-risk, and low-risk. HR; hazard ratio, DFS; disease-free survival, CI; 95% confidence interval. (TIF) [file pone.0123946.s008.tif]

**FIGURE S2**

**A**

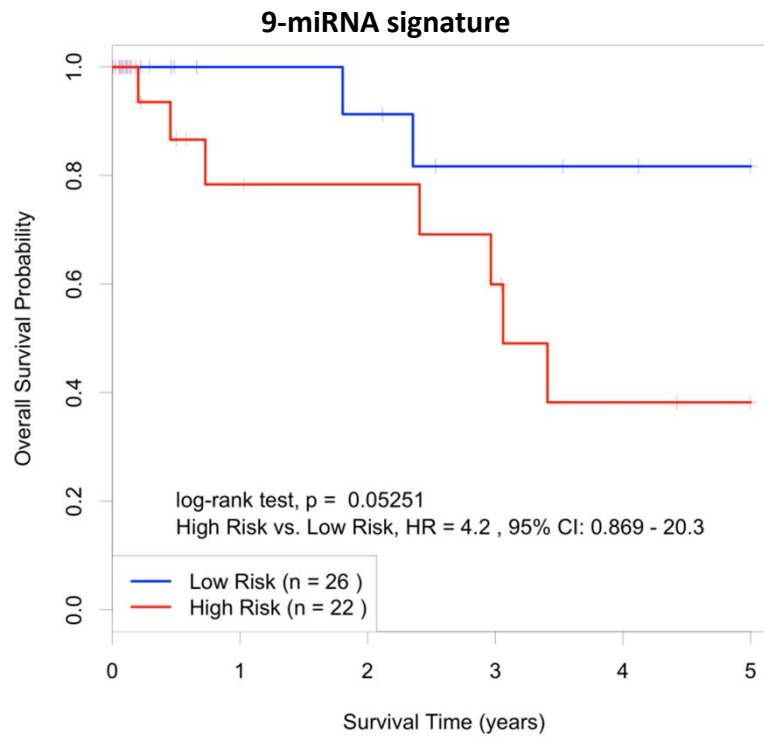

**B**

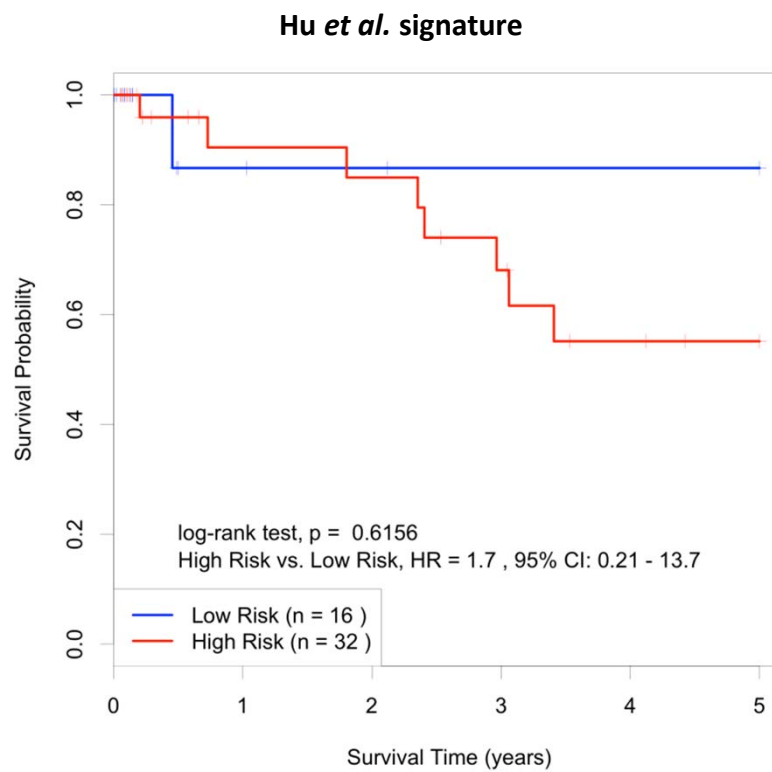

Supplement: S2 Fig — miRNA expression data from the TCGA miRNASeq cohort (n = 48) was used to test: A) our 9-miR signature, and B) the Hu et al. 2-miR signature. HR; hazard ratio, CI; 95% confidence interval. (PDF) [file pone.0123946.s009.pdf]
